# Essential Medicines Availability, Pricing, and Stock-Outs for Hypertension and Diabetes in Private Retail Pharmacies in Zimbabwe

**DOI:** 10.3390/ijerph23020215

**Published:** 2026-02-09

**Authors:** Laston Gonah, Sibusiso Cyprian Nomatshila, Sikhumbuzo Advisor Mabunda, Wilson Wezile Chitha

**Affiliations:** 1 School of Public Health, Faculty of Medicine and Health Sciences, Walter Sisulu University, Mthatha 5100, South Africa; snomatshila@wsu.ac.za (S.C.N.); smabunda@wsu.ac.za (S.A.M.); wchitha@wsu.ac.za (W.W.C.); 2 WSU Society and Health Research Institute, Walter Sisulu University, Mthatha 5100, South Africa; 3 Global Centre for Human Resources for Health Intelligence, Walter Sisulu University, East London 5247, South Africa; 4 School of Population Health, University of New South Wales, Sydney 2052, Australia; 5 WSU Institute for Clinical Governance and Healthcare Administration, Faculty of Medicine and Health Sciences, Walter Sisulu University, Mthatha 5100, South Africa

**Keywords:** essential medicines, stock-outs, medicine availability, medicine pricing, diabetes mellitus, hypertension, human capital productivity, private pharmacies, urban Zimbabwe

## Abstract

**Highlights:**

**Public health relevance—How does this work relate to a public health issue?**

- Hypertension and diabetes are major contributors to morbidity and mortality in Zimbabwe, yet reliable access to essential medicines remains a critical health system-wide challenge.
- Private retail pharmacies increasingly serve as a key alternative source of chronic disease medicines amid recurrent public-sector stock-outs.

**Public health significance—Why is this work of significance to public health?**

- This study provides empirical evidence on medicine availability, pricing, and stock-out patterns in private pharmacies, addressing a major evidence gap in the Zimbabwean NCD medicine landscape.
- The findings highlight substantial price disparities between private retail markets, public facilities, and international reference prices, revealing potential affordability barriers for patients with NCDs.

**Public health implications—What are the key implications or messages for practitioners, policy makers and/or researchers in public health?**

- High and variable retail medicine prices, combined with the high prevalence of HTN–DM comorbidity, can significantly increase out-of-pocket spending and jeopardize long-term treatment adherence.
- Strengthening pricing regulation and optimizing supply-chain processes are essential policy priorities to ensure equitable access to essential NCD medicines in urban private-sector markets.

**Abstract:**

**Background**: Access to affordable essential medicines is critical for effective management of hypertension (HTN) and diabetes mellitus (DM). In Zimbabwe, frequent stock-outs in public facilities position private pharmacies as important alternative sources of these medicines. **Aim:** To assess availability, pricing, and stock-out levels of essential HTN and DM medicines in private retail pharmacies in Gweru Urban District, Zimbabwe. **Methods:** A cross-sectional survey was conducted in 40 registered private pharmacies. Data on medicine availability, retail prices, monthly stock-outs, and supply-chain factors were collected using a structured interviewer-administered questionnaire, stock cards, and observational checklists. Local prices were compared with international reference prices (IRPs). Chi-square analyses evaluated associations between pharmacy characteristics, medicine prices, availability, and stock-out durations. **Results:** Most tracer medicines for HTN and DM were available in ≥80% of pharmacies, with average stock-outs generally <3 days per month. Pharmacy characteristics were not significantly associated with availability or stock-outs. Medicines with <80% availability and those priced at ≥USD 5 were significantly associated with prolonged stock-outs of ≥7 days (*p* = 0.006 and *p* = 0.001, respectively). Local retail prices exceeded IRPs and public facility prices, suggesting potential affordability barriers in the context of an economic crisis, where most health expenditures are out-of-pocket. Key drivers of stock-outs included wholesaler shortages, delivery delays, limited procurement funds, and substitution with alternative medicines. **Conclusions:** While medicine availability and short-term stock-outs were generally favourable, high retail prices pose a major potential barrier to access. The cost burden is amplified by the common HTN-DM comorbidity, requiring multiple medications per person, thereby further increasing out-of-pocket expenses. High prices may limit adherence, reduce functional capacity, and negatively impact productivity. Policy interventions targeting pricing regulations and value-chain optimization are urgently needed to enhance equitable access to essential NCD medicines in urban Zimbabwe.

## 1. Introduction

Access to essential medicines is central to effective health system performance and population health. Yet, consistent availability remains a major challenge in many low- and middle-income countries (LMICs), where the World Health Organization (WHO) estimates that up to half of the population lacks reliable access to essential medicines [1,2]. Sub-Saharan Africa (SSA) carries a dual burden of infectious diseases and an escalating prevalence of non-communicable diseases (NCDs), notably hypertension (HTN) and diabetes mellitus (DM), which require lifelong adherence to therapy [3]. Effective management of these conditions is vital not only for health outcomes but also for safeguarding human capital and economic productivity.

WHO defines essential medicines as those that should always be available, in adequate quantities, appropriate dosage forms, and at prices affordable to individuals and communities [4]. However, public health facilities across SSA frequently experience prolonged stock-outs, especially for NCD medicines. In many LMICs, up to 90% of people rely on out-of-pocket expenditure to obtain medicines [4,5,6,7], making medicines the second-largest household expense after food [8]. Consequently, affordability significantly influences treatment adherence.

Since many NCDs are risk factors for one another, individuals often experience comorbidity or multimorbidity, for example, the frequent coexistence of HTN and DM [9,10]. Patients living with multiple chronic conditions therefore require several medicines concurrently, compounding treatment costs and increasing the likelihood of catastrophic health expenditure [9,10,11]. In contexts where public-sector supplies are unreliable, private retail pharmacies serve as a primary source of NCD medicines, making availability and pricing crucial determinants of access.

Zimbabwe’s experience with HIV demonstrates how consistent access to essential medicines, in this case, antiretroviral therapy (ART), can yield excellent health outcomes, including high viral suppression and increased life expectancy [11]. However, people living with HIV (PLHIV) face an increased risk of NCDs, partly attributable to ageing and long-term ART exposure [5,11]. Alongside the rising NCD prevalence, driven by behavioural and lifestyle factors [12], demand for HTN and DM medicines continues to grow.

Previous studies in Zimbabwe indicate that public facilities frequently experience stock-outs of HTN and DM medicines, prompting patients to seek treatment from private pharmacies, where care is predominantly funded out-of-pocket [13,14]. Despite growing evidence on essential medicine availability in public health facilities in Zimbabwe and other LMICs, limited empirical data exist on the dynamics of availability, pricing, and stock-out duration within the private retail pharmacy sector, particularly in medium-sized urban settings. Existing studies have largely focused on public-sector supply constraints or assessed availability without integrating pricing and stock-out duration metrics [8,14,15].

This study addresses this gap by providing an integrated assessment of essential medicines for HTN and DM in private retail pharmacies in Gweru. By simultaneously examining availability, stock-out duration, pricing relative to international benchmarks and public-sector prices, and pharmacy-reported supply-chain constraints, the study offers policy-relevant insights into private-sector performance within a mixed health system under economic instability. This study was designed as a descriptive assessment of availability, pricing benchmarks, and stock-out patterns in private pharmacies, rather than a comprehensive affordability or policy impact evaluation. The findings contribute new evidence on how private pharmacies function as a critical, yet potentially inequitable, access point for chronic disease medicines, with direct implications for pricing regulation, value-chain optimization, and NCD policy planning in urban LMIC contexts.

## 2. Methodology

### 2.1. Research Design

A cross-sectional descriptive study employing quantitative data collection and analysis was conducted.

### 2.2. Study Setting

The study was conducted in all private retail pharmacies in Gweru Urban District, Midlands Province, Zimbabwe, where 96% of pharmacies are located in the central business district. These pharmacies serve urban populations as well as surrounding rural areas with limited access to private pharmacies.

### 2.3. Study Population and Sampling

A total of 50 registered private retail pharmacies were identified in Gweru Urban District. Seven newly established pharmacies (operational for less than six months) were excluded due to insufficient operational history, and three pharmacies were excluded after participating in pre-testing of the data collection tool. The final analytical sample therefore comprised 40 pharmacies.

### 2.4. Data Collection

Primary variables included availability of essential medicines for HTN and DM, number of stock-out days per month, local retail prices compared with WHO International Reference Prices (IRPs) [16], and factors influencing stock-outs. Tracer medicines were selected based on the EDLIZ 8th Edition guidelines [17] and local prescribing patterns, reflecting commonly used first-line and alternative agents. Tracers were not weighted by prescribing frequency but were included to capture real-world private-sector availability.

The selected tracer medicines included hypertensive medications such as diuretics (hydrochlorothiazide, indapamide, spironolactone, furosemide), angiotensin-converting enzyme inhibitors (lisinopril, enalapril, captopril), angiotensin receptor blockers (losartan), beta blockers (atenolol, propranolol, metoprolol, bisoprolol), calcium channel blockers (amlodipine, nifedipine), vasodilators (hydralazine, prazosin), centrally acting agents (methyldopa), and combination therapies (Urazide, Exforge, Tenoric) and diabetic medications, including insulins (short-acting, intermediate, long-acting), biguanides (metformin), sulfonylureas (glibenclamide, gliclazide, glimipride), and dipeptidyl peptidase IV (DPP-IV) inhibitors (vildagliptin) [17]. For all tracer medicines, specific strengths and quantities were determined to represent a one-month supply for data collection and analysis.

Availability on the survey day was verified through observation, while stock-outs were obtained from records.

Data collection used a structured, interviewer-administered questionnaire composed of validated questions [13,18] that was pre-tested in three private retail pharmacies. The instrument was revised based on pre-test findings, and pharmacies involved in the pre-test were excluded from participation in the main study. Availability of medicines on the day of the survey was verified using an observational checklist, while stock-out, defined as the number of days each medicine was unavailable within a month, were obtained from stock cards and dispensing records. Factors influencing stock-outs were reported by pharmacy owners or managers through the questionnaire.

IRPs for all tracer medicines were obtained from the 2015 Management Sciences for Health (MSH) IRP Indicator Guide [16], with local prices collected in 2021. While more recent IRPs were unavailable, these served as standardized benchmarks, although noting that inflation and currency instability may affect precise price comparisons. This time gap is acknowledged as a potential source of temporal bias in the comparison. Local median retail prices in private pharmacies (2021) were compared with public sector facility prices from the same year and with 2015 IRPs to assess relative price differentials. Although IRPs were not contemporaneous with local prices and comparisons should be interpreted with caution, they were used soley as standardized international benchmarks for relative price comparison, not as direct measures of affordability or precise cost ratios.

### 2.5. Data Presentation and Analysis Procedures

Data were entered and analyzed using IBM SPSS Version 29^®^. Descriptive statistics were used to summarize the characteristics of each study variable. Categorical variables, including the availability of tracer medicines and reported reasons for stock-outs, were expressed as frequencies and percentages, while continuous variables, such as average stock-out days across facilities and local medicine prices, were assessed for normality. Normally distributed continuous variables were summarized using means and standard deviations, whereas non-normally distributed variables were summarized using medians and interquartile ranges.

Medicine availability was calculated as the percentage of pharmacies with each tracer medicine in stock on the day of the survey, while stock-out levels were averaged across pharmacies for the number of days medicines were unavailable in a month. Medicine availability was dichotomized using the WHO threshold, where availability in ≥80% of pharmacies was considered adequate, while availability in <80% of pharmacies indicated suboptimal access [1,2,4]. Mean stock-out durations were categorized as low (≤3 days per month), moderate (4–6 days), and prolonged (≥7 days), consistent with previous studies on medicine availability in LMIC settings [8,18,19]. Median local retail prices for all tracer medicines were compared against WHO IRPs [16], and percentage differences were calculated to assess pricing deviations. Chi-square analyses evaluated associations between pharmacy characteristics, medicine prices, availability, and stock-out durations.

Responses from pharmacy owners or managers regarding factors affecting medicine availability were summarized using frequencies and percentages. Key findings were presented in tables, bar charts, and figures to illustrate trends in medicine availability, stock-outs, and pricing differences, facilitating clear visual comparisons across tracer medicines.

### 2.6. Ethical Considerations

Written informed consent was obtained from all participants, and data were anonymized and securely stored. Ethical approval was waived as the study primarily relied on pharmacy records and observational data, with minimal direct involvement of human participants.

## 3. Results

### 3.1. Overall Response Rate

A total of 40 questionnaires were administered to 40 private retail pharmacies, with responses provided by pharmacy owners or managers. All questionnaires were completed, corresponding to a 100% response rate, allowing comprehensive assessment of essential medicine availability, stock-outs, pricing, and factors influencing non-availability across the study sites.

### 3.2. Availability of Essential Medicines

**Figure 1:**
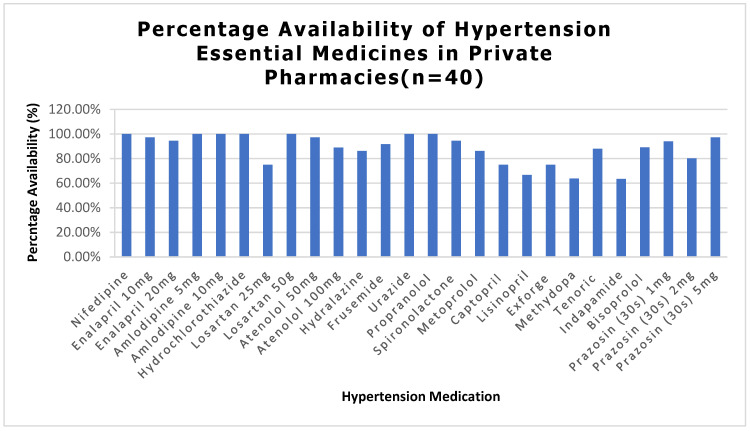
Percentage Availability of HTN Essential Medicines in Private Pharmacies.

**Figure 2:**
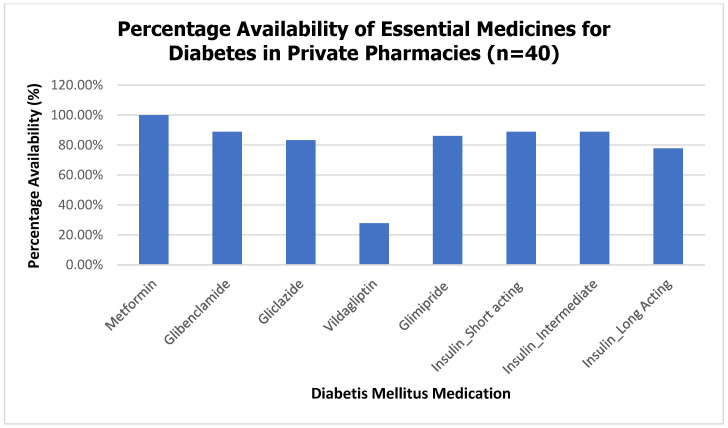
Percentage Availability of Essential Medicines for DM in Private Pharmacies.

Availability of essential medicines was assessed on the day of the survey. Most tracer hypertensive medicines were widely available, with 64% to 100% of pharmacies stocking each medicine and an overall average availability of 88.6% (Figure 1). DM medications showed slightly lower availability, ranging from 28% to 100% across tracer medicines, with an overall average availability of 80.2% (Figure 2).

### 3.3. Stock-Outs of Essential Medicines

Stock-outs were measured as the number of days a medicine was unavailable within a one-month period. Among hypertensive medicines, six out of twenty classes had no recorded stock-outs. Most of the remaining medicines (19 out of 26) recorded an average of less than 7 stock-out days per month, corresponding to low-to-moderate stock-out levels, with an overall mean of 1.5 days (SD ± 2.83).

Among DM medicines, most products (six of eight) experienced low-to-moderate stock-outs of fewer than seven days per month. Two products had notably longer periods of non-availability, with average stock-out durations of 13 and 29 days. The overall mean stock-out duration across all DM products was 7.4 days per month (SD ± 9.63), indicating variability in stock-out patterns between products. Vildagliptin had the longest average stock-out duration at 29 days (SD ± 3.71), while most other DM medicines recorded stock-outs of fewer than seven days (Table 1).

### 3.4. Pricing of Essential Medicines

Information on the prices of tracer essential medicines was collected from all 40 pharmacies. Median local prices were compared with the MSH IRPs from 2015 [16] and 2021 public facility United States Dollar (USD$) prices.

Across all private pharmacies, inter-pharmacy price variation for essential HTN medicines was low at 5.9%, while diabetes medicines showed a similarly small variation of 4.7%, indicating that prices were largely uniform across pharmacies for most formulations. Overall, medicines priced ≥$5 were more likely to have monthly availability below 80% and stock-outs lasting ≥7 days. Local median retail prices were substantially higher than international reference prices (IRPs) and prices in public facilities, highlighting potential affordability barriers (Table 2).

For example, the median local price of hydrochlorothiazide in private pharmacies was US$1.11 compared to the IRP of US$0.71, while metformin was US$2.04 versus an IRP of US$0.49. Insulins and newer medications, such as vildagliptin, showed the largest discrepancies indicating a possible significant financial burden for patients accessing essential medicines in private retail pharmacies in Gweru urban district.

Further analysis comparing local private pharmacy prices to IRPs [16] confirmed that all 23 essential medicine classes assessed were priced higher than international benchmarks (Table 3 and Table 4).

**Average Comparison of all Selected Products with Data:**

- Buyer Price: 833.89% (0);
- Prices in (currency): USD$.

**Average Comparison of all Selected Products with Data:**

- Buyer Price: 1402.73% (0);
- Prices in (currency): USD$.

### 3.5. Factors Influencing Stock-Outs

Chi-square analyses indicated that medicine availability, dichotomised according to the WHO-recommended threshold of ≥80% versus <80% of pharmacies stocking a given medicine [1], was significantly associated with the duration of stock-outs. Medicines with <80% availability across pharmacies were more likely to experience prolonged stock-outs of ≥7 days (*p* = 0.006) for both HTN and DM medicines.

When retail price was categorized into two groups (<USD 5 vs. ≥USD 5), both medicine availability and stock-out duration were significantly associated with price. Medicines priced at ≥USD 5 were more likely to have monthly availability below 80% (*p* = 0.001) and to experience prolonged stock-outs lasting ≥7 days (*p* = 0.00013), compared with lower-priced medicines. Other pharmacy characteristics, including years of operation and location within the central business district, were not statistically associated with medicine availability or stock-out outcomes (*p* > 0.05).

**Figure 3:**
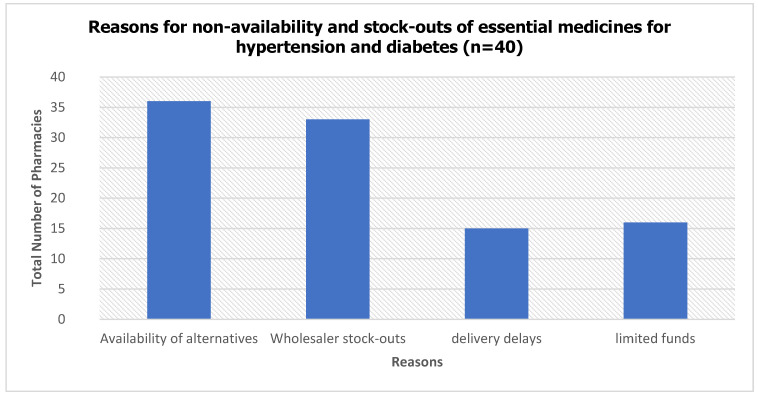
Reported Reasons for Non-availability and Stock-outs of Essential Medicines for Hypertension and Diabetes.

Commonly reported reasons (from owners’ and managers’ perspectives) for low availability and prolonged stock-outs of both HTN and DM tracer medicines were predominantly supply-chain-related, including wholesaler stock-outs, delivery delays and supplier reliability issues, limited pharmacy purchasing capacity, and formulation-specific logistics or substitution with alternative medicines (Figure 3). Multiple factors were often reported per facility, highlighting the complex interplay of financial and logistical constraints affecting medicine availability. Reported factors influencing availability and stock-outs for HTN and DM medicines were largely similar, with no additional reasons specified for injectable products such as insulin. Data collection did not specifically probe potential differences between oral and injectable medicines, which may have obscured formulation-specific barriers to availability and stock-outs.

Although the survey was conducted during the COVID-19 pandemic, the pandemic itself was not cited by respondents as a major perceived cause of stock-outs, suggesting that similar availability challenges may have existed prior to COVID-19 and may persist beyond the pandemic period.

## 4. Discussion

This study provides insights into availability, stock-outs, pricing, and determinants of essential medicines for HTN and DM in private pharmacies in Gweru. Overall, availability was high, stock-out low, but retail prices exceeded IRPs and public facility prices. Main factors influencing stock-outs were alternative medicines, wholesaler shortages, delivery delays, and limited procurement funds.

Availability of essential medicines in private pharmacies was generally high. Similarly, stock-out levels were low, with the majority of medicines unavailable for fewer than three days per month. These findings are consistent with previous studies conducted in Zimbabwe and other LMICs, which report higher availability and lower stock-out rates in private pharmacies compared to public facilities [6,13,18].The World Health Organization (WHO) recommends a minimum essential medicines availability threshold of ≥80% to ensure adequate access within a defined geographical area [1,4]. High availability and low stock-out levels in private pharmacies suggest that patients may have better chances of accessing essential medicines when public sector stocks are unavailable.

However, high availability alone does not guarantee access. Although affordability was not assessed in this study, it remains a critical factor influencing whether patients can consistently access essential treatments, particularly given the high poverty levels and the predominance of out-of-pocket health expenditures in Zimbabwe. [4,5,6]. Despite generally high availability, medicines for HTN and DM were priced substantially above IRPs and local public sector rates. Importantly, data collection occurred during the COVID-19 pandemic, a period characterized by global supply disruptions, transport constraints, and heightened economic instability, which may have exacerbated procurement challenges and pricing pressures in the private sector [20], while the absence of affordability assessments represent a major limitation of this study. The observed availability patterns therefore reflect private-sector performance under stress conditions. However, despite gradual post-pandemic recovery, many structural constraints identified, such as import dependence, currency volatility, and high out-of-pocket financing, remain relevant, highlighting the continued policy significance of these findings [14,21,22,23].

Private retail pharmacy prices were significantly higher than both IRPs and public facility prices, likely due to a combination of procurement costs, overheads, taxes, regulatory fees, and market-driven pricing strategies. In Zimbabwe, where poverty levels remain high [23,24,25], these elevated prices may hinder consistent access to essential medicines, particularly for chronic conditions such as HTN and DM that require long-term therapy. The financial burden is further compounded when patients require multiple medications due to comorbidities, which is common given that HTN and DM often coexist and increase the risk of other NCDs [5,6,12].

Previous studies have shown that private pharmacy prices in Zimbabwe can be up to ten times higher than regional averages and IRPs [9,13,25]. In this study, local retail prices substantially exceeded international reference benchmarks, indicating potential affordability challenges, although the precise magnitude should be interpreted cautiously given the unmeasured possible inflation and currency instability. While public facilities offer lower prices, these are still above international benchmarks, yet most patients rely on out-of-pocket payments, thereby increasing the risk of interrupted treatment [6,13,25]. This has implications not only for individual health outcomes but also for public health and economic productivity, as uncontrolled HTN and DM may lead to premature morbidity, mortality, and reduced human capital.

Respondents identified multiple reasons for non-availability and stock-outs, including wholesaler stock-outs, delays in delivery, limited funds for procurement, and the availability of alternative medicines. The presence of alternative medicines can influence both prescribing and dispensing patterns. For instance, medicines such as lisinopril and losartan were less frequently available (<80%), whereas nifedipine, amlodipine, and enalapril, often used as alternatives, showed higher availability. Pharmacy ordering practices are frequently influenced by local prescribing trends, patient demand, and supplier recommendations, shaping observed stock-out patterns [2,5,6,7].

Wholesaler stock-outs and delivery delays are likely compounded by Zimbabwe’s dependence on imported active pharmaceutical ingredients and finished products [21,22,25,26]. Additional contextual factors, including the economic disruptions caused by the COVID-19 pandemic, unstable exchange rates, and high operational costs, though not assessed in this study, may have exacerbated procurement challenges contributing to temporary stock-outs.

Limited funding for procurement also plays a role. Private pharmacies must balance overheads, salaries, taxes, and regulatory fees while managing fluctuating exchange rates, which can restrict timely re-stocking of essential medicines [13,14,23,25,26]. While this study did not quantify the impact of taxation and regulatory fees, these factors likely contribute to both high prices and periodic stock-outs.

### 4.1. Best Practices for Pricing of Essential Medicines

Pricing strategies for essential medicines vary by country and context, including competition-based, cost-based, and reference price-based approaches [15,27]. The study findings highlight the need for strategies to enhance affordability while ensuring continued supply. Potential best practices for Zimbabwe include pooled procurement or bulk purchasing through a central agency, such as the National Pharmaceutical Company (NatPharm), for both public and private sectors. This could reduce procurement costs and establish standard pricing thresholds.

A review of the essential medicines value chain is also recommended to identify cost drivers. Policy interventions might include relaxing import restrictions, reducing taxes and regulatory fees for essential medicines, and implementing government-industry negotiations to regulate prices. Such strategies could improve affordability and contribute to equitable access.

### 4.2. Limitations

This study has several limitations that should be considered when interpreting the findings. Availability was measured on a single day, while stock-outs were assessed retrospectively over one month, which may not fully capture temporal fluctuations in medicine supply. The study included only 40 urban private pharmacies, limiting generalizability to rural areas or the broader province. Affordability was not directly measured; while local prices were compared with 2015 IRPs and contemporaneous public sector prices, no more recent benchmarks were available, and currency instability and inflation may affect interpretation. The study did not conduct sensitivity analyses or explore alternative reference points, which could have strengthened the robustness of price comparisons. Data collection did not specifically probe potential differences between oral and injectable formulations, such as cold chain or handling requirements, and did not assess the specific impacts of COVID-19 on supply chains, which may have masked formulation-specific or pandemic-related barriers to availability and stock-outs. Despite these constraints, the study provides valuable insights into private sector medicine availability, pricing, and supply chain challenges in a medium-sized urban setting, with implications for policy and practice in similar contexts.

### 4.3. Recommendations

Based on the study findings, the following recommendations are proposed:

1. Researchers and public health professionals should conduct larger, multi-site studies across urban and rural areas to generate generalizable evidence on the availability, stock-outs, and pricing of essential medicines in private pharmacies in Zimbabwe.
2. Health policymakers and regulatory authorities must implement longitudinal monitoring systems to track medicine availability, stock-outs, and price trends over time, providing a more comprehensive understanding of supply patterns.
3. Health economists and policymakers must assess medicine affordability by comparing treatment costs with household income or minimum wage data to identify barriers to access and inform pricing policies.
4. Pharmaceutical procurement and supply chain actors must explore pooled procurement or bulk purchasing mechanisms to reduce procurement costs, stabilize supply, and improve affordability of essential medicines in private pharmacies.
5. Policy and regulatory stakeholders must review the essential medicines value chain to identify cost drivers, including import restrictions, taxes, and regulatory fees, and implement strategies such as price negotiations with suppliers to reduce retail prices.

## 5. Conclusions

This study found that availability of essential medicines for HTN and DM in private retail pharmacies in Gweru urban district was generally satisfactory, with most tracer medicines observed to be in stock and short-term stock-outs occurring infrequently. However, despite adequate availability, high retail prices may represent a major potential barrier to access. This financial burden can be further compounded by the frequent co-occurrence of HTN and DM, which often requires the concurrent use of multiple medicines, thereby increasing out-of-pocket expenditure. Elevated medicine costs may limit long-term treatment adherence, reduce functional capacity, and affect productivity, with broader implications for human capital and economic outcomes. Overall, while private retail pharmacies may play an important role in maintaining medicine availability, affordability may remain a critical challenge to equitable access to NCD medicines in urban Zimbabwe. Policy interventions targeting pricing, supply-chain optimization, and strategic procurement may help improve affordability and support more sustainable access to essential medicines.

## Figures and Tables

**Table 1:** Essential Medicines Stock-outs and Availability for HTN and DM.

Drug Name	Number of Pharmacies Experiencing Stock-Outs (n)	Average Stock-Outs Days per Month (Mean ± SD)	Median Local Price (Mean ± SD)	Median IRP (Buyer Price) [16]
**HTN medicines**				
Hydrochlorothiazide	0	0	$1.11 (0.21)	$0.71
Nifedipine	2	0 (0.2323)	$2.78 (0.48)	$1.38
Enalapril 10 mg Enalapril 20 mg	4 1	0 (0.8819) 0 (0.6666)	$2.42 (0.47) $3.26 (0.51)	$0.31 $0.34
Amlodipine 5 mg Amlodipine 10 mg	0 2	0 0 (0.5225)	$4.38 (0.67) $5.18 (0.54)	$0.18 $0.27
Losartan 25 mg Losartan 50 mg	5 2	1 (1.8385) 0 (0.5225)	$5.26 (0.49) $3.58 (0.44)	- $0.54
Atenolol 50 mg Atenolol 100 mg	0 3	0 0 (0.5426)	$2.75 (0.35) $2.60 (0.43)	$0.17 $0.26
Hydralazine inj 1 vial	13	1 (2.2608)	$7.79 (0.45)	$2.23
Furosemide	1	0 (0.3333)	$1.60 (0.41)	$1.04
Urazide	0	0	$2.61 (0.42)	-
Propranolol	1	0 (0.5)	$1.96 (0.25)	$1.09
Spironolactone	11	2 (2.8884)	$4.58 (0.46)	$1.33
Metoprolol	15	1 (1)	$17.88 (0.57)	$1.33
Captopril	24	3 (2.5898)	$2.60 (0.43)	$0.23
Lisinopril	26	1 (1.2860)	$8.64 (0.44)	$0.70
Exforge	38	9 (3.3858)	$35.67 (0.76)	-
Methydopa	34	5 (2.1014)	$24.83 (0.53)	$1.40
Tenoric	33	6 (2.5127)	$8.92 (0.33)	-
Indapamide	40	10 (2.1396)	$11.11 (0.57)	-
Bisoprolol	0	0	$6.51 (0.53)	$1.39
Prazosin 1 mg Prazosin 2 mg Prazosin 5 mg	0 3 0	0 0 (0.2803) 0	$3.33 (0.46) $5.78 (0.39) $5.31 (0.47)	- - -
**DM medicines**				
Insulins - Short-acting - Intermediate - Long-acting	36 34 36	5 (1.5) 3 (1.9475) 13 (3.3701)	$8.15 (0.57) $8.15 (0.57) $16.65 (0.89)	$3.88 $3.88 -
Metformin	0	0	$2.04 (0.18)	$0.49
Glibenclamide	3	0 (1.0173)	$1 (0)	$0.16
Gliclazide	34	5 (1.8263)	$5.17 (0.49)	$0.98
Vildagliptin	36	29 (3.7105)	$13.14 (0.35)	-
Glimipride	35	4 (1.3809)	$14.06 (0.72)	$0.26

**Table 2:** Comparisons of Essential Medicines Prices * in Private Pharmacies, Public Facilities, and IRPs.

Drug Name	Average Drug Price in Private Pharmacies (Mean ± SD)	Average Public Sector Price	IRPs (Buyer Price) [16]
**HTN medicines**			
Hydrochlorothiazide (HCT), 30 s	$1.11 (0.21)	$0.76	$0.71
Nifedipine, 30 s	$2.78 (0.48)	$2.30	$1.38
Enalapril 20 mg, 30 s	$3.26 (0.51)	$2.30	$0.34
Amlodipine 10 mg, 30 s	$5.18 (0.54)	$2.30	$0.27
Losartan 50 mg, 30 s	$3.58 (0.44)	$2.30	$0.54
Atenolol 50 mg, 30 s	$2.75 (0.35)	$1.54	$0.17
Furosemide 30 s	$1.60 (0.41)	$0.76	$1.04
Urazide 30 s	$2.61 (0.42)	$1.54	-
Captopril 30 s	$2.60 (0.43)	$2.30	$0.23
Methydopa 180 s	$24.83 (0.53)	$3.08	$1.40
**DM medicines**			
Insulins - Short acting - Intermediate - Long acting	$8.15 (0.57) $8.15 (0.57) $16.65 (0.89)	$1.54 $1.54 $1.54	$3.88 $3.88 -
Metformin 30 s	$2.04 (0.18)	$0.76	$0.49
Glibenclamide, 30 s	$1 (0)	$0.76	$0.16

* Prices are expressed in United States dollars (USD), the legal tender in Zimbabwe during the study period.

**Table 3:** **International Medical Products Price Guide—Management Sciences for Health (MSH).** Source: MSH Price Guide—Buyer prices, https://msh.org/resources/international-medical-products-price-guide-2/; (Author account; accessed on 30 April 2021). Compare My Prices: My Drug List Title: Selected Essential Medicines for Hypertension.

Product Name	Strength	Dosage Form	Package Quantity	Current Price (US$)	Buyer Median	Buyer Price (% of Buyer Median)
Nifedipine	20 mg	TABLET OR CAPSULE	30 TAB-CAP(s)	2.78	0.0461/Tab-cap (1)	201.01%
Enalapril	20 mg	TABLET OR CAPSULE	30 TAB-CAP(s)	3.26	0.0114/Tab-cap (2)	953.22%
Enalapril	10 mg	TABLET OR CAPSULE	30 TAB-CAP(s)	2.42	0.0102/Tab-cap (3)	790.85%
Amlodipine	5 mg	TABLET OR CAPSULE	30 TAB-CAP(s)	4.38	0.0061/Tab-cap (4)	2393.44%
Amlodipine	10 mg	TABLET OR CAPSULE	30 TAB-CAP(s)	5.18	0.0092/Tab-cap (4)	1876.81%
Hydrochlorothiazide	25 mg	TABLET OR CAPSULE	30 TAB-CAP(s)	1.11	0.0237/Tab-cap (4)	156.12%
Losartan	50 mg	TABLET OR CAPSULE	30 TAB-CAP(s)	3.58	0.0181/Tab-cap (4)	659.30%
Atenolol	50 mg	TABLET OR CAPSULE	30 TAB-CAP(s)	2.75	0.0059/Tab-cap (3)	1553.67%
Atenolol	100 mg	TABLET OR CAPSULE	30 TAB-CAP(s)	2.60	0.0085/Tab-cap (1)	1019.61%
Hydralazine	20 mg	AMPOULE	1 AMPOULE(s)	7.79	2.2290/Ampoule (4)	349.48%
Furosemide	10 mg/mL	AMPOULE	50 mL(s)	1.60	0.0662/mL (5)	48.34%
Propranolol HCl	10 mg	TABLET OR CAPSULE	30 TAB-CAP(s)	1.96	0.0365/Tab-cap (2)	179.00%
Spironolactone	25 mg	TABLET OR CAPSULE	30 TAB-CAP(s)	4.58	0.0442/Tab-cap (5)	345.40%
Metoprolol	100 mg	TABLET OR CAPSULE	30 TAB-CAP(s)	17.88	0.0444/Tab-cap (1)	1342.34%
Captopril	25 mg	TABLET OR CAPSULE	30 TAB-CAP(s)	2.60	0.0076/Tab-cap (4)	1140.35%
Lisinopril	20 mg	TABLET OR CAPSULE	30 TAB-CAP(s)	8.64	0.0233/Tab-cap (3)	1236.05%
Methyldopa	250 mg	TABLET OR CAPSULE	180 TAB-CAP(s)	24.83	0.0467/Tab-cap (5)	295.38%
Bisoprolol	5 mg	TABLET OR CAPSULE	30 TAB-CAP(s)	6.51	0.0462/Tab-cap (1)	469.70%

**Table 4:** **International Medical Products Price Guide—Management Sciences for Health (MSH).** Source: MSH Price Guide—Buyer prices, https://msh.org/resources/international-medical-products-price-guide-2/; (Author account; accessed on 30 April 2021). Compare My Prices: My Drug List Title: Selected Essential Medicines for Diabetes Mellitus.

Product Name	Strength	Dosage Form	Package Quantity	Current Price (US$)	Buyer Median	Buyer Price (% of Buyer Median)
Metformin HCl	500 mg	TABLET OR CAPSULE	30 TAB-CAP(s)	2.04	0.0162/Tab-cap (2)	419.75%
Glibenclamide	5 mg	TABLET OR CAPSULE	30 TAB-CAP(s)	1.00	0.0053/Tab-cap (4)	628.93%
Gliclazide	30 mg	TABLET OR CAPSULE	30 TAB-CAP(s)	5.17	0.0325/Tab-cap (1)	530.26%
Glimepiride	2 mg	TABLET OR CAPSULE	30 TAB-CAP(s)	14.06	0.0087/Tab-cap (1)	5386.97%
Insulin, Isophane	100 IU/mL	VIAL	100 mL(s)	16.65	0.3488/mL (4)	47.74%

## Data Availability

The data analyzed during the current study is available from the corresponding author [Laston Gonah, Email: lgonah@wsu.ac.za] on reasonable request to bona fide researchers.
